# Systematic family‐wide analysis of sodium bicarbonate cotransporter NBCn1/SLC4A7 interactions with PDZ scaffold proteins

**DOI:** 10.14814/phy2.12016

**Published:** 2014-05-20

**Authors:** Hye Jeong Lee, Min Hyung Kwon, Soojung Lee, Randy A. Hall, C. Chris Yun, Inyeong Choi

**Affiliations:** 1 Department of Pediatrics, Division of Hematology and Oncology, Vanderbilt University, Nashville, Tennessee, USA; 2 Department of Physiology, Emory University School of Medicine, Atlanta, Georgia, USA; 3 Department of Pharmacology, Emory University School of Medicine, Atlanta, Georgia, USA; 4 Department of Medicine, Division of Digestive Disease, Emory University School of Medicine, Atlanta, Georgia, USA

**Keywords:** Bicarbonate transporter, pH, protein interaction, SLC4A7, *Xenopus* oocyte

## Abstract

NBCn1 (SLC4A7) plays a role in transepithelial HCO_3_^−^ movement and intracellular pH maintenance in many tissues. In this study, we searched PDZ proteins capable of binding to NBCn1. We screened a protein array membrane, on which 96 different class I PDZ protein peptides were blotted, with the C‐terminal domain of NBCn1 fused to GST. Thirteen proteins were identified in these screens: MAGI‐3, NHERF‐1, NHERF‐2, PSD‐95, chapsyn‐110, ERBIN, MALS‐1, densin‐180, syntrophins *α*1, *β*2, *γ*2, MUPP1, and PDZK1. After determining these binding partners, we analyzed the database of known and predicted protein interactions to obtain an NBCn1 interaction network. The network shows NBCn1 being physically and functionally associated with a variety of membrane and cytosolic proteins via the binding partners. We then focused on syntrophin *γ*2 to examine the molecular and functional interaction between NBCn1 and one of the identified binding partners in the *Xenopus* oocyte expression system. GST/NBCn1 pulled down syntrophin *γ*2 and conversely GST/syntrophin *γ*2 pulled down NBCn1. Moreover, syntrophin *γ*2 increased intracellular pH recovery, from acidification, mediated by NBCn1's Na/HCO_3_ cotransport. Syntrophin *γ*2 also increased an ionic conductance produced by NBCn1 channel‐like activity. Thus, syntrophin *γ*2 regulates NBCn1 activity. In conclusion, this study demonstrates that NBCn1 binds to many PDZ proteins, which in turn may allow the transporter to associate with other physiologically important proteins.

## Abstract (precis)

e12016

We identified proteins capable of binding to the sodium/bicarbonate cotransporter NBCn1/SLC4A7 and built the NBCn1 interaction network. The data show that NBCn1 physically and functionally associates with a variety of proteins.

## Introduction

NBCn1 (SLC4A7) is a member of SLC4 bicarbonate transporters that include Cl/HCO_3_ exchangers, Na/HCO_3_ cotransporters, and Na^+^‐driven Cl/HCO_3_ exchangers (Choi 2012; Romero et al. 2013). NBCn1 normally moves Na^+^ and HCO_3_^−^ into cells and protects intracellular pH (pH_i_) from falling below normal. The transporter also plays a role in transepithelial HCO_3_^−^ movement in epithelial tissues where HCO_3_^−^ secretion/absorption affects cellular and systemic acid‐base homeostasis. In addition, the transporter has intrinsic channel‐like activity that primarily produces Na^+^ conductance (Choi et al. 2000; Cooper et al. 2005; Lee et al. 2012). NBCn1 shares high amino acid sequence with other bicarbonate transporters (Boron et al. 2009); nonetheless its function, cellular localization, and physiological significance are different from others (Choi 2012). Genetic mutation and gene polymorphism studies show that abnormalities in NBCn1 are pathologically associated with visual and hearing defects (Bok et al. 2003), vascular muscle contractility and hypertension (Boedtkjer et al. 2011), and breast cancer (Boedtkjer et al. 2012; Lauritzen et al. 2012).

NBCn1 interacts with other proteins. In the kidney, NBCn1 binds to the 56 kDa subunit of H‐ATPase and tethers with H‐ATPase on the same side of the membrane (Pushkin et al. 2003). In the pancreatic ducts and salivary glands, NBCn1 binds to the Na/H exchanger regulatory factor‐1 (NHERF‐1/EBP50) and modulates luminal HCO_3_^−^ secretion by Cl channels (Park et al. 2002). In the ears, NBCn1 binds to harmonin that is a constituent of a macromolecular complex in hair cells (Reiners et al. 2005). In the brain, NBCn1 binds to the postsynaptic density protein PSD‐95 (Lee et al. 2012) that interacts with many different ion channels, receptors, and signaling proteins (Dosemeci et al. 2007). The binding mechanism involves the interaction of the NBCn1 C‐terminal last four amino acid residues with PDZ domains in the binding partners (PDZ: *p*ost synaptic density protein, *D*rosophila disk large tumor suppressor, and *z*onula occludens‐1 protein) (Kim and Sheng 2004). The last amino acid sequence in NBCn1 is E‐T‐S‐L, which is conserved in all NBCn1 splice variants. Taken together, these studies reveal that different PDZ proteins can bind to NBCn1 to alter transporter activity, formation of macromolecular complexes, and other cellular processes in tissue‐specific manners.

In this study, we performed a systematic family‐wide analysis in order to gain a more panoramic view of the set of PDZ proteins capable of binding to NBCn1. The C‐terminal domain of NBCn1 was used as a probe to screen a protein array membrane on which different PDZ domains were blotted. Thirteen interactions were identified, some of which have been previously reported as NBCn1‐binding partners and some of which are novel. We also examined molecular and functional interactions of NBCn1 and syntrophin *γ*2 in *Xenopus* oocytes. This study shows that NBCn1 binds to a variety of membrane‐associated PDZ proteins and that the novel interaction with syntrophin *γ*2 has functional consequences for NBCn1 activity.

## Materials and Methods

### Ethical issues

All experiments in this study were conducted under the NIH guidelines for research on animals, and experimental protocols were approved by the Institutional Animal Care and Use Committee at Emory University.

### PDZ filter overlay assay

Screening the PDZ protein array was performed according to published protocols (He et al. 2006; Zhang et al. 2007). The Glutathione S‐Transferase (GST) fusion protein containing the C‐terminal 26 amino acids of rat NBCn1 was prepared as described previously (Lee et al. 2012). A nylon membrane, on which 96 different Class I PDZ domains were spotted in a rectangular grid (Zhang et al. 2007), was preincubated in blot buffer (50 mmol/L NaCl, 2% dry milk, 0.1% Tween 20, 10 mmol/L HEPES; pH 7.4) for 30 min at room temperature. The membrane was then incubated with 100 nmol/L GST/NBCn1 fusion proteins in fresh blot buffer for 1 h. The control was incubation with GST only. After washes, the membrane was then incubated with the horse radish peroxidase (HRP)‐conjugated anti‐GST antibody (Merck Millipore; Billerica, MA) for 1 h. GST/NBCn1 fusion proteins overlaid onto the array were detected by the ECL chemiluminescence kit (GE Healthcare; Piscataway, NJ). Experiments were done three times.

### Protein interaction network

The interactions between NBCn1 and identified binding partners were analyzed using the Search Tool for the Retrieval of Interacting Genes/Proteins STRING (version 9.0; http://www.string-db.org), a search tool of known and predicted protein interactions based on physical and functional associations (Jensen et al. 2009). All 13 proteins identified were used for analysis. Harmonin and the 56 kDa H‐ATPase subunit were not included in the search because harmonin was not detected in our array experiment and the molecular entity the 56 kDa subunit remains unclear although it could be the B2 subunit. For search parameters, the active prediction method was set to experiment, which shows a list of protein interaction datasets gathered from multiple databases; the required confidence score was set to 0.4 (medium confidence); and the interactors shown was set to no more than 50. The organism was set to human. The network view was displayed in an interactive mode in which nodes could be freely moved. The edges represent the predicted functional associations and are drawn with up to seven differently colored lines: activation (green), inhibition (red), binding (blue), phenotype (cyan), catalysis (brown), posttranslational modification (purple), reaction (black), and expression (yellow). Other marks are described in the website help section. The image of the network was exported to Microsoft powerpoint, and thick black lines between NBCn1 and 13 proteins were added to indicate physical interactions.

### GST pull‐down assay

GST/NBCn1 fusion proteins were constructed by subcloning rat NBCn1 (Genbank accession number NM_058211) nucleotides into pGEX‐4T vector (GE Healthcare). The C‐terminal 131 amino acids of rat NBCn1 was fused to GST. The GST/syntrophin *γ*2 fusion protein was made by subcloning the human syntrophin *γ*2 (Piluso et al. 2000) into pPEX‐4T. GST/Syn‐full contained the full length syntrophin *γ*2 (amino acid residues 1‐540). GST/Syn‐PDZ contains amino acids 1–231 of syntrophin *γ*2 and thus includes the PDZ domain (aa 73–156). GST/Syn‐ΔPDZ contains amino acids 232–540 and thus lacks the PDZ domain but includes the peckstrin homology PH domain (aa 296‐421). GST fusion proteins and GST only were purified from bacteria using Glutathione‐Sepharose 4B beads (GE Healthcare) according to the manufacturer's protocol and resuspended in phosphate‐buffered saline (PBS) containing 0.5% Nonidet P‐40 and 1× protease inhibitor cocktail (Merck Millipore). The equal amounts of GST/fusion proteins or GST only bound to beads were then incubated with lysates of oocytes or rat brains at 4°C with gentle rotation for 4 h. After incubation, beads were washed five times with ice–cold PBS containing 0.5% Nonidet P‐40 and bound proteins were dissociated from the beads by adding the SDS‐PAGE sample loading buffer. Proteins were loaded on a 7.5% SDS polyacrylamide gel, separated, and blotted to a nitrocellulose membrane. The blot was incubated with the rabbit NBCn1 antibody (1:500 dilution) or rabbit syntrophin *γ*2 antibody (1:1000) for 2 h in PBS containing 0.05% Tween 20 and 5% nonfat dry milk. The blot was washed and incubated with the HRP‐conjugated anti‐rabbit IgG (Millipore) for 1 h, and immunoreactive bands were visualized using the ECL kit.

### Protein expression in *Xenopus* oocytes

Female frogs were purchased from Xenopus Express (Brooksville, FL). A frog was anesthetized with fresh 0.1% 3‐aminobenzoic acid ethyl ester for 20 min and surgery was done to collect oocytes. After suture, the frog was returned to a recovery tank containing 0.1 mol/L NaCl. Oocytes were washed in a solution (in mmol/L; 96 NaCl, 2 KCl, 1 MgCl_2_, and 10 HEPES, pH 7.5) five times for 20 min each, and follicles were removed with type IA collagenase (2 mg/mL; Sigma‐Aldrich, St. Louis, MO) twice for 20 min each. Stage V‐VI oocytes were sorted and stored in OR3 medium at 18°C overnight. For injection, NBCn1 and syntrophin *γ*2 were in vitro transcribed using the mMessage/mMachine transcription kit (Life Technologies; Grand Island, NY) and cRNAs were injected at 20 ng NBCn1 per oocyte (in 40 nL) and 2, 10, and 20 ng syntrophin *γ*2 per oocyte. Oocytes were maintained at 18°C for 3 days before use. For membrane preparation, oocytes were homogenized with a syringe with 23G needle in the homogenization buffer (300 mmol/L mannitol, 2 mmol/L EDTA, 1 mmol/L phenylmethanesulfonyl fluoride, 5 mmol/L HEPES) with 1× protease inhibitor cocktail. Cell debris was removed by centrifugation at 3000 *g* for 10 min, and membrane lysates were obtained by centrifugation at 100,000 *g* for 1 h at 4°C. The protein concentration was determined using the Bradford reagents (Sigma‐Aldrich).

### Measurement of pH_i_ in oocytes

An oocyte was impaled with a pH electrode containing the proton ionophore 1 cocktail B (Sigma‐Aldrich) backfilled with the phosphate buffer at pH 7.0. The pH electrode was connected to the high‐impedance electrometer FD‐223 (World Precision Instruments; Sarasota, FL) and then routed to a custom‐made subtraction amplifier. The second electrode was a voltage electrode containing 3 mol/L KCl to monitor oocyte membrane potentials. The voltage electrode was connected to an OC‐725C oocyte clamp amplifier (Warner Instrument; Hamden, CT). The bath electrode connected to the amplifier served as a reference electrode. Signals from pH and voltage electrodes were sampled by a Digidata 1322A interfaced to a computer. Data acquisition was performed using pClamp (Molecular Devices, Sunnyvale, CA). A pH electrode signal was subtracted from a voltage electrode signal to calculate a voltage for pH. The slope of voltage to pH was determined using pH 6.0 and 8.0 standards, which was typically at the range of 53 ± 3 mV/pH. For pH recording, an oocyte was superfused with ND96 solution (in mmol/L; 96 NaCl, 2 KCl, 1.8 CaCl_2_, 1 MgCl_2_, and 10 HEPES, pH 7.4) and then with a solution buffered with 25 mM HCO_3_^−^, 5% CO_2_ (pH 7.4). NaCl was replaced mole for mole with NaHCO_3_. The rate of pH_i_ recovery (dpH_i_/dt) from CO_2_‐induced acidification was determined by linear regression after pH_i_ reached the lowest point. Experiments were done at room temperature.

### Measurement of NBCn1 conductance

The ionic conductance mediated by NBCn1 was recorded by two‐electrode voltage clamp. Glass capillaries with 1 mm outer diameter were filled with 3 mol/L KCl and had resistances of 1–2 ΩM. The two electrodes were connected to voltage and current probes which were connected to an OC‐725C. Signals from electrodes were sampled by a Digidata 1322A, and data acquisition was done by pClamp. An oocyte was clamped at −60 mV in ND96 solution and a step voltage was commanded from −140 to +40 mV with 20‐mV increments (100 ms duration for each increment). An inwardly rectifying current at negative potentials with the slope conductance of > 4 *μ*S was considered as an NBCn1 current. A slope was measured near a zero‐current potential. The conductance was also confirmed by frequently recording in 0 mmol/L Na^+^, which reduces the slope by more than 50% (Cooper et al. 2005). Experiments were done in the nominal absence of HCO_3_^−^.

### Statistical analysis

Data were reported as means ± standard error. Levels of significance were assessed using the unpaired, two‐tailed Student's *t*‐test. The *P*‐value of less than 0.05 was considered significant.

## Results

### Class I PDZ proteins capable of binding to NBCn1

**Figure 1.**
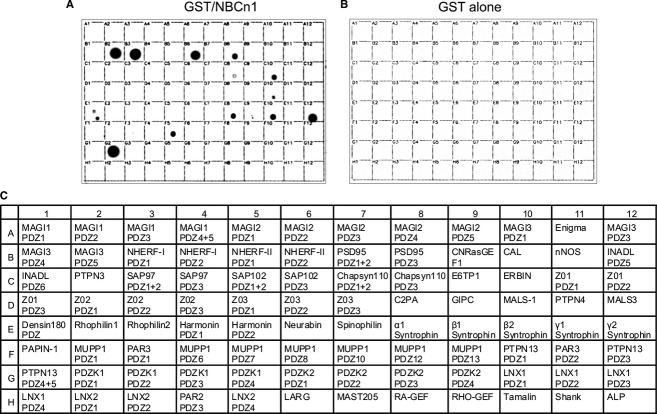
A proteomic analysis to identify PDZ proteins capable of binding to NBCn1. (A) A proteomic array containing 96 class I PDZ domains from 55 different proteins was screened with the C‐terminal domain of NBCn1 fused to GST. The domains bound to GST/NBCn1 were detected by the anti‐GST antibody. (B) The control experiment with GST only. (C) The PDZ domains spotted on the membrane are listed. The proteins binding to NBCn1 are MAGI‐3 (B2 in the array), NHERF‐1 (B3), NHERF‐2 (B6), PSD‐95 (B8), chapsyn‐110 (C8), ERBIN (C10), MALS‐1 (D10), densin‐180 (E1), syntrophins *α*1 (E8), *β*2 (E10), *γ*2 (E12), MUPP1 (F5), and PDZK1 (G2).

We screened a protein array membrane, on which 96 different PDZ domains from 55 proteins were dot blotted (Fig. 1C), with the GST/NBCn1 fusion protein containing the C‐terminal 26 amino acids of rat NBCn1. The proteins bound to GST/NBCn1 were then detected by the GST antibody. Figure 1A shows the results: MAGI‐3 (PDZ domain 5), NHERF‐1 (domain 1), NHERF‐2 (domain 2), PSD‐95 (domain 3), chapsyn‐110 (domains 1 and 2), ERBIN, MALS‐1, densin‐180, syntrophins *α*_1_, *β*_2_ and *γ*_2_, MUPP1 (domain 7), and PDZK1 (domain 1). MAGI‐3, NHERF‐I, NHERF‐II, and PDZK1 showed strong signals, suggesting that these proteins have relatively strong biochemical interactions with NBCn1. MALS‐1 and densin‐180 showed weaker signals. The identification of NHERF‐1 and PSD‐95 as binding partners is consistent with the previous reports on their interactions with NBCn1 in native tissues or heterologous systems determined by colocalization or coimmunoprecipitation (Park et al. 2002; Lee et al. 2012). As for PSD‐95, the third PDZ domain (residue 307–446; B8) was recognized by GST/NBCn1, whereas the first and second domains (residue 59‐303; position B7) were not recognized. Our assay did not recognize harmonin (E4 and E5) that was reported to bind to NBCn1 in photoreceptor cells (Reiners et al. 2005). The reason for this discrepancy remains unclear. The control experiment with GST only showed no detection (Fig. 1B).

### NBCn1 protein interaction network

**Figure 2.**
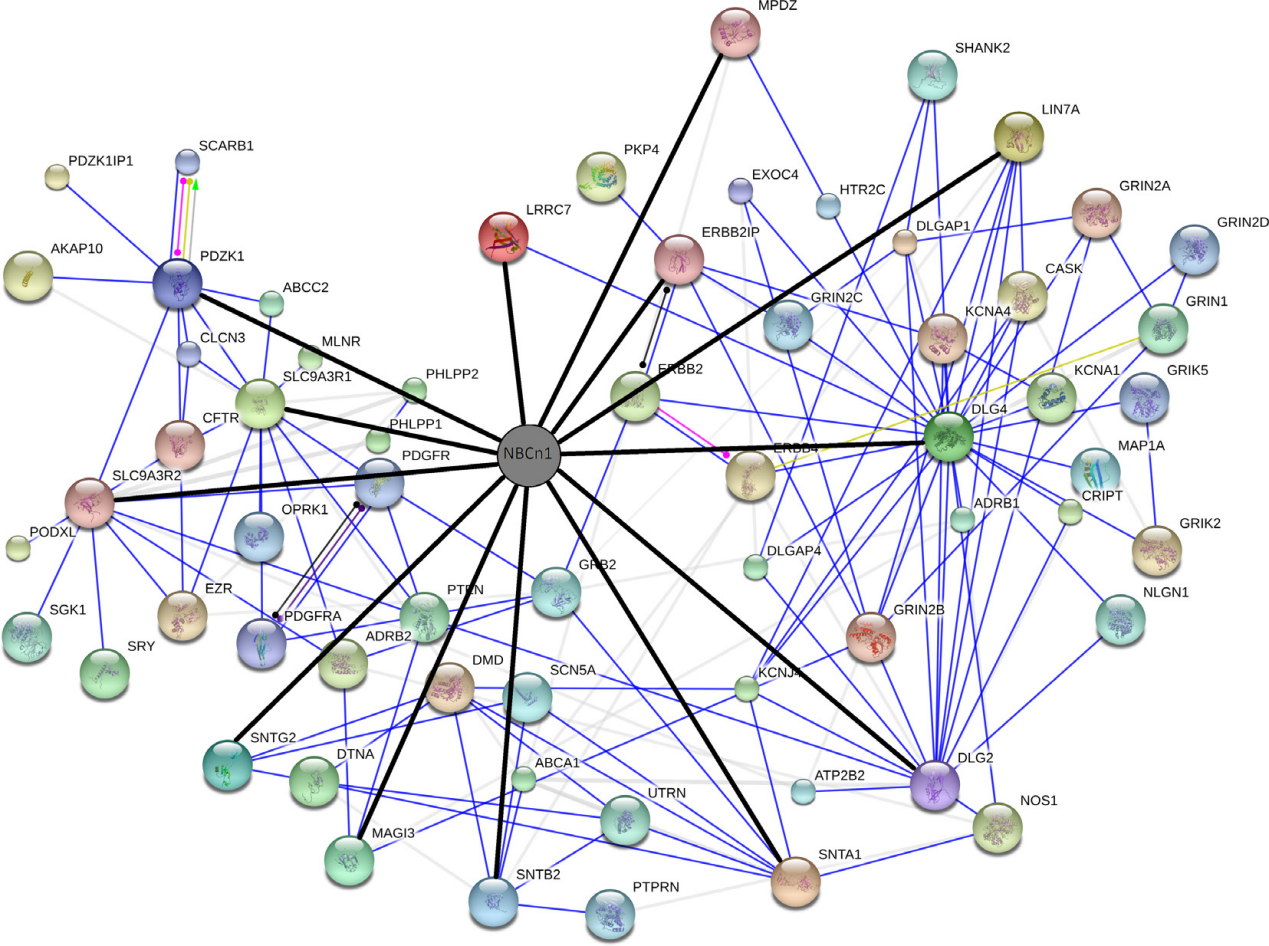
NBCn1 interaction network. The interaction network was built using the STRING program that searches known and predicted protein interactions based on physical and functional associations. The search parameters are described in the Materials and Methods. The interactions between NBCn1 and identified binding proteins (*thick black lines*) are supported from the array experiments, and the interactions involving individual binding proteins (*blue lines*) are drawn from experiments‐based evidence in the database. In addition, physical and functional associations among proteins are shown (*differently colored lines*). Proteins were named according to the human gene/protein name.

With the information on proteins capable of binding to NBCn1, we then analyzed the NBCn1 interaction network using the STRING program that searches known and predicted protein interactions based on physical and functional associations. Figure 2 shows the result displayed in the network view. The interactions between NBCn1 and 13 binding proteins (thick black lines in the figure) are supported from our array data, and interactions involving individual binding proteins (blue lines) are drawn from experiments‐based evidence (such as affinity chromatography) available in the database. In addition, physical and functional associations among proteins are also demonstrated (differently colored lines). The interaction network shows NBCn1 associations with a variety of membrane and cytosolic proteins via identified binding proteins. The associations of NBCn1 with the cystic fibrosis transmembrane conductance regulator CFTR, plasma membrane calcium ATPase ATP2B2, and *N*‐methyl‐d‐glutamate receptors GRINs confirm previous reports or suggestions (Park et al. 2002; Bok et al. 2003; Lee et al. 2012). The NBn1‐binding proteins and their association proteins are summarized in Table 1.

**Table 1.** NBn1‐binding proteins and their association proteins.

PDZ protein (domain)	Human gene name	Array ID	UniProt access ID	Major interacting proteins
MAGI‐3 (5)	MAGI3	B2	Q5TCQ9	PTEN, USP43, TNK2, VANGL2, PTPRB, FZD4
NHERF‐I (1)	SLC9A3R1	B3	O14745	CFTR, EZR, ADRB2, MLNR, PTEN, OPRK1
NHERF‐II (2)	SLC9A3R2	B6	Q15599	CFTR, PODXL, PHLPP1, PHLPP2, SRY, SGK1
PSD‐95 (3)	DLG4	B8	P78352	GRIN2B, GRIN2A, KCNA4, DLGAP1, GRIK2, ERBB4
Chapsyn‐110 (1 & 2)	DLG2	C8	Q15700	GRIN2B, ABCA1, DLGAP4, GRIN2A, ATP2B2, NOS1
ERBIN	ERBB2IP	C10	Q96RT1	PKP4, ERBB2, SMAD3, SMAD2, RPAP1, LRRC1
MALS‐1	LIN7A	D10	O14910	CASK, MPP6, KCNJ12, AMOT, ABCA1, SLC6A12
Densin‐180	LRRC7	E1	Q01HU8	CAMK2G, CNKSR2, TOR1A, KSR2, CTNND2, DLG4
Syntrophin *α*1	SNTA1	E8	Q13424	DMD, DTNA, ABCA1, GRB2, SCN5A, NOS1
Syntrophin *β*2	SNTB2	E10	Q13425	UTRN, PTPRN, ABCA1, MARK2, DTNA, MAST2
Syntrophin *γ*2	SNTG2	E12	Q9NY99	SCN5A, DTNB, DTNA, DMD
MUPP1 (7)	MPDZ	F5	O75970	HTR2C, PLEKHA2, PLEKHA1, AMOT, MAGEB18, SYNGAP1
PDZK1 (1)	PDZK1	G2	Q5T2W1	PDZK1IP1, AKAP10, CFTR, ABCC2, SCARB1, SLC22A4

### Interaction between NBCn1 and syntrophin γ2

**Figure 3.**
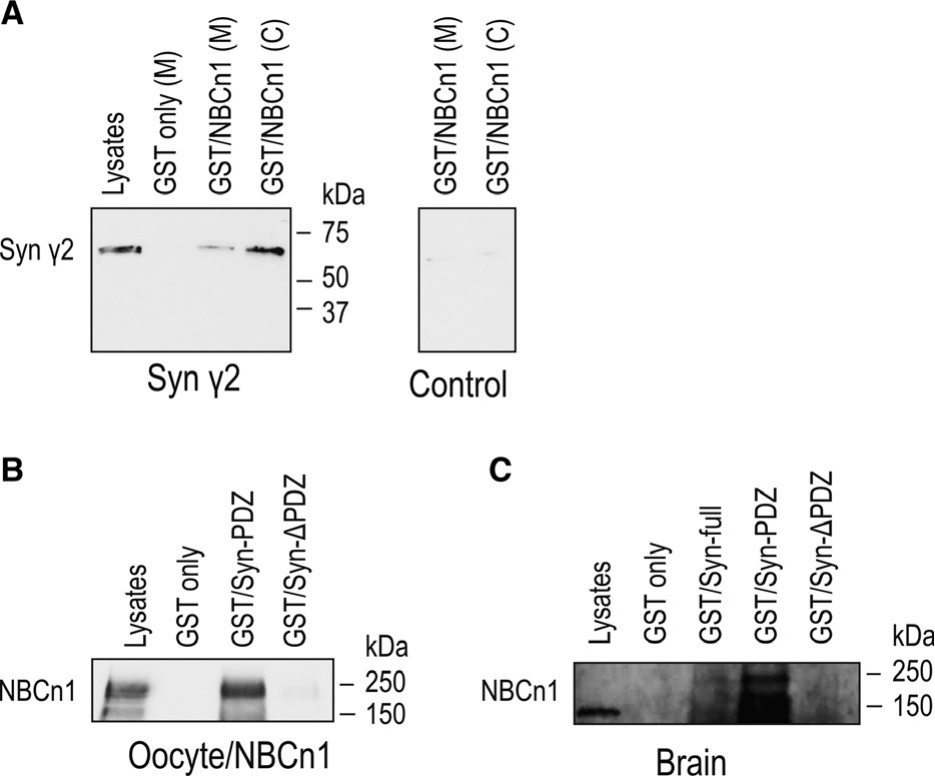
Interaction between NBCn1 and syntrophin *γ*2. (A) Lysates of *Xenopus* oocytes expressing syntrophin *γ*2 or none were incubated with GST/NBCn1 fusion proteins containing the C‐terminal amino acids of NBCn1. GST only served as a control. Pull‐down samples were immunoblotted with the syntrophin *γ*2 antibody. Syntrophin *γ*2 (60 kDa) was detected in pull–down samples from syntrophin *γ*2‐expressing oocytes, but not from control oocytes. Lysates were prepared from membrane (M) and cytosol (C). (B) Lysates of oocytes expressing rat NBCn1 were incubated with GST only, GST/Syn‐PDZ containing amino acid residues 1‐231 of syntrophin *γ*2, which include the PDZ domain, and GST/Syn‐ΔPDZ containing residues 232–539, which include the peckstrin homology domain. NBCn1 was pulled down by GST/Syn‐PDZ, but not by GST/Syn‐ΔPDZ. One of three experiments is shown. (C) Membrane lysates of rat brains were incubated with GST only, GST/Syn‐full containing the full‐length syntrophin *γ*2, GST/Syn‐PDZ, or GST/Syn‐ΔPDZ. NBCn1 was pulled down by GST/Syn‐full and GST/Syn‐PDZ, but not by GST/Syn‐ΔPDZ.

We next focused on syntrophin *γ*2 to examine the molecular and functional interaction between NBCn1 and one of the novel binding partners identified in our screens. Syntrophin *γ*2 was chosen because, unlike other NBCn1‐binding proteins, it is highly localized to the endoplasmic reticulum and is proposed to play a primary role in trafficking proteins (Alessi et al. 2006). This feature is advantageous for our examining NBCn1 function, particularly testing whether channel‐like activity and Na/HCO_3_ cotransport of NBCn1 can be separated by a trafficking molecule. The two properties can be separated by PSD‐95 as PSD‐95 stimulates NBCn1 channel‐like activity without affecting Na/HCO_3_ cotransport (Lee et al. 2012). For this analysis, we first determined the physical interaction between NBCn1 and syntrophin *γ*2 in the *Xenopus* oocyte expression system. Figure 3A shows the results from pull‐down assays in which membrane and cytosolic lysates of oocytes expressing syntrophin *γ*2 were incubated with GST/NBCn1 or GST only and pull‐down products were immunoblotted with the syntrophin *γ*2 antibody. Syntrophin *γ*2 (60 kDa) was detected in pull‐down samples, supporting the interaction between NBCn1 and syntrophin *γ*2. The signal intensity was stronger in the cytosolic sample, in good agreement with predominant subcellular localization of syntrophin *γ*2 (Alessi et al. 2006).

We then performed reciprocal experiments in which NBCn1 was expressed in oocytes and pulled down by GST/syntrophin *γ*2 (Fig. 3B). For this analysis, we constructed, GST/Syn‐PDZ containing amino acid residues 1‐231 of syntrophin *γ*2, which include the PDZ domain (residues 73–156), and GST/Syn‐ΔPDZ containing residues 232–539, which include the PH domain (residues 296–421). Immunoblotting with the NBCn1 antibody revealed that NBCn1 in oocyte membranes was pulled down by GST/Syn‐PDZ, but not by GST/Syn‐ΔPDZ. A typical 150 kDa band and a ~250 kDa band were detected in both lysates and pull‐down products. The 250 kDa band is probably an aggregate of the transporter that is often detected in oocyte samples under the denatured condition. In other experiments, we performed pull‐down assays using rat brain lysates (Fig. 3C). NBCn1 was pulled down by GST/Syn‐full that contained the full‐length syntrophin γ2 (amino acid residues 1‐540) and GST/Syn‐PDZ, but not by GST/Syn‐ΔPDZ, thus consistent with the results from oocytes expressing NBCn1.

### Increased NBCn1 activity by syntrophin γ2

**Figure 4.**
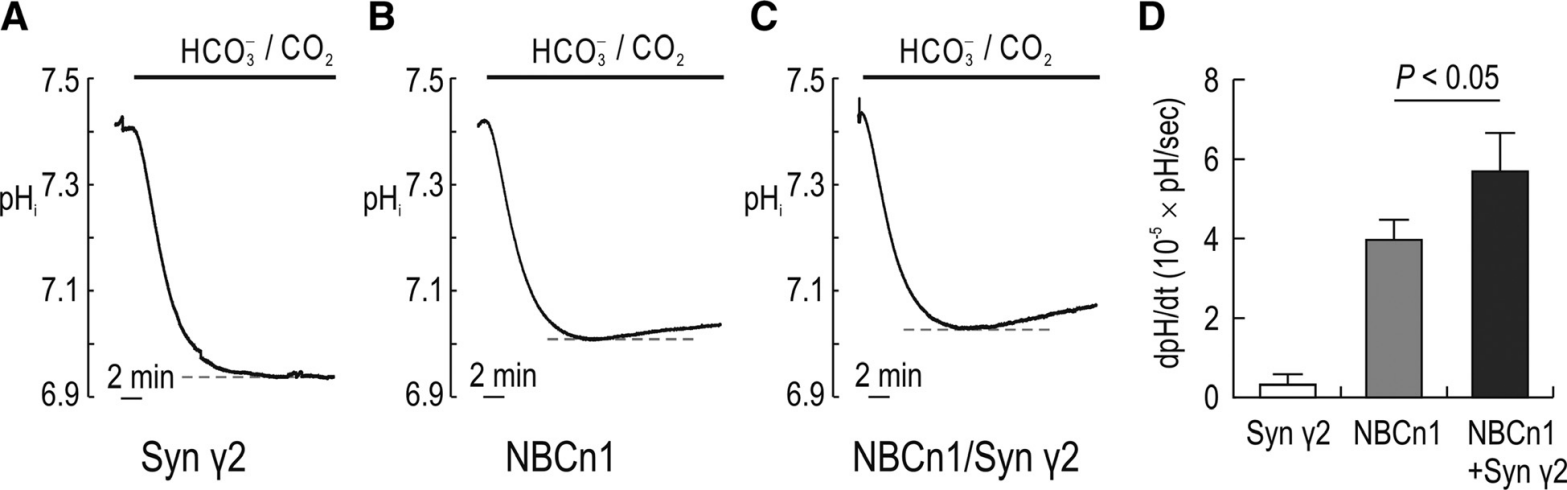
Effect of syntrophin *γ*2 on NBCn1‐mediated pH_i_ recovery in *Xenopus* oocytes. (A–C) Representative pH_i_ traces in oocytes expressing syntrophin *γ*2, NBCn1, and NBCn1/syntrophin *γ*2. Oocytes were superfused with HCO_3_^−^/CO_2_‐free ND96 solution and then with a solution equilibrated with 25 mmol/L HCO_3_^−^, 5% CO_2_. pH_i_ was measured with a proton‐selective glass electrode. (D) Mean pH_i_ recovery rate. The recovery rate (dpH_i_/dt) was calculated from a linear regression line during the first 2 min of recovery (*n *= 5 for each).

To assess the effect of syntrophin *γ*2 on NBCn1 function, we measured pH_i_ of oocytes using a proton‐selective microelectrode. NBCn1 moves HCO_3_^−^ into oocytes and raises pH_i_, and thus we measured pH_i_ recovery from an intracellular acidification in the presence of HCO_3_^−^/CO_2_. Figure 4 shows representative pH_i_ traces in oocytes. In an oocyte expressing syntrophin *γ*2 (Fig. 4A), applying 25 mmol/L HCO_3_^−^, 5% CO_2_ (pH 7.4) caused pH_i_ to fall as CO_2_ entered, was hydrated, and produced H^+^. The pH_i_ then remained at a steady‐state pH as intracellular CO_2_ was equal to extracellular CO_2_. This confirms that syntrophin *γ*2 alone does not induce pH_i_ recovery from CO_2_‐induced intracellular acidification. In an oocyte expressing NBCn1 (Fig. 4B), the pH_i_ was recovered from acidification as HCO_3_^−^ transported via NBCn1 compensated intracellular H^+^. The compensation also reduced CO_2_‐induced acidification. In an oocyte coexpressing NBCn1 and syntrophin *γ*2 (Fig. 4C), the pH_i_ was recovered at a higher rate than that for NBCn1 only. Figure 4D summarizes mean recovery rates (dpH_i_/dt) calculated during the first 2 min of recovery (*n *=**5 for each). The rate was greater in NBCn1/syntrophin *γ*2 than in NBCn1 only (5.75 ± 0.5 × 10^−5^ for NBCn1/syntrophin *γ*2 vs. 3.97 ± 0.2 × 10^−5^ pH units/sec for NBCn1; *P *<**0.05).

**Figure 5.**
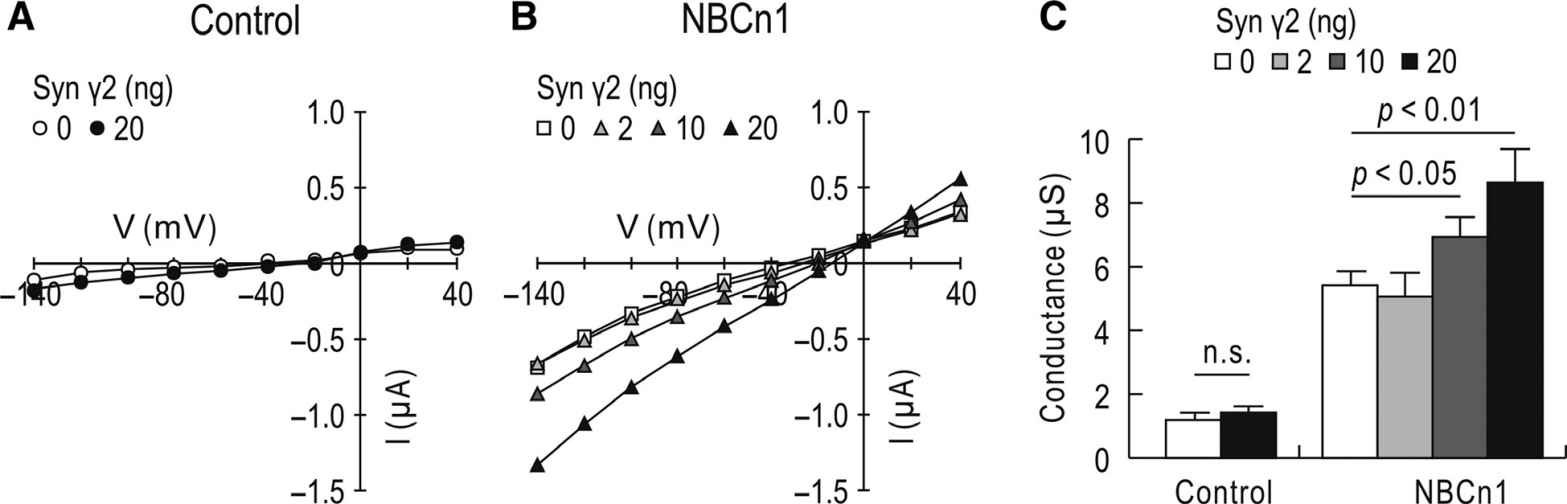
Effect of syntrophin *γ*2 on NBCn1 conductance. (A) Representative current–voltage (*I–V*) relationships in a syntrophin *γ*2‐expressing oocyte or an uninjected oocyte. Oocytes were clamped at −60 mV and subjected to the voltage command stepping from −140 to +40 mV. Steady‐state currents were recorded (*n *=**5 for each). Recordings were done in HCO_3_^−^/CO_2_‐free ND96 solution. (B) *I–V* relationships in oocytes expressing NBCn1 and different amounts of syntrophin *γ*2. Large inward currents at negative potentials and outward currents at positive potentials are hallmarks for NBCn1 conductance. (C) Mean slope conductance. Slopes were determined near zero‐current voltages in *I–V* plots. Data were averaged from uninjected control oocytes (*n *=**12), oocytes expressing syntrophin *γ*2 (*n *=**16), NBCn1 (*n *=**15), NBCn1 plus syntrophin *γ*2 at 2 ng (*n *=**17), 10 ng (*n *=**15), and 20 ng (*n *=**24). n.s, not significant. *P*‐values were calculated to compare each NBCn1/syntrophin with NBCn1 only.

We then measured NBCn1‐mediated ionic conductance by two‐electrode voltage clamp. Current–voltage (*I–V*) relationships were determined by applying a stair‐case voltage command from −140 to 40 mV with the holding potential of −60 mV. Figure 5A and B show representative *I–V* relationships. An uninjected control oocyte produced negligible basal currents and similarly an oocyte expressing syntrophin *γ*2 (20 ng cRNAs per oocyte) had negligible basal currents. In contrast, an oocyte expressing NBCn1 produced distinct inward currents at negative potentials and distinct outward currents at positive potentials, hallmarks for the channel‐like activity of NBCn1. The currents were progressively larger at higher amounts of injected syntrophin cRNAs and the slopes were raised according to the change in the currents. Figure 5C summarizes mean slope conductance measured at the voltage with zero current. The slope conductance was increased at 10 ng and 20 ng of syntrophin *γ*2 (5.39 ± 0.21 *μ*S for NBCn1, *n *=**15; 6.89 ± 0.68 *μ*S for NBCn1 plus 10 ng syntrophin *γ*2, *n *=**15; 8.63 ± 1.08 for NBCn1 plus 20 ng syntrophin *γ*2, *n *=**24; *P *<**0.05). The conductance was unaffected at 2 ng of syntrophin *γ*2, probably due to insufficient amount of the protein.

## Discussion

The major finding from this study is the identification of a large set of membrane‐associated proteins capable of binding to NBCn1. Except for NHERF‐I and PSD‐95, most of the NBCn1‐binding partners identified in our screens represent novel associations. Moreover, in addition to providing a panoramic view of the set of NBCn1 interactions with proteins, our studies also shed light on the specific PDZ domains serving as the structural determinants for the interactions. The identification of these binding partners led us to build the NBCn1 interaction network that comprises a variety of ion channels and receptors, scaffolding proteins, cytoskeleton, signaling and regulatory proteins. The results from functional studies of the NBCn1/syntrophin *γ*2 interaction support the idea that the binding proteins can change NBCn1 activity. Overall, this study sheds significant light on the NBCn1 interaction network that offers a foundation for future studies of the physiological significance of these various interactions.

The identification of NBCn1‐binding proteins in this study is based on the biochemical interaction between the NBCn1 C‐terminal domain and PDZ protein peptides blotted on a nitrocellulose membrane. While it remains unclear whether all of the identified proteins interact with NBCn1 in native cells, their cellular and subcellular localization provide some valuable insight. In neurons, PSD‐95 (Dosemeci et al. 2007), chapsyn‐110/PSD‐93 (Kim et al. 1996), ERBIN (Borg et al. 2000), densin‐180 (Apperson et al. 1996), and MUPP1 (Estevez et al. 2008) are localized to postsynaptic membranes, where NBCn1 is highly expressed (Park et al. 2010; Lee et al. 2012). Other proteins including NHERF‐2 (Paquet et al. 2006), syntrophin *α*1 (Gorecki et al. 1997), MALS‐1/Lin7A (Misawa et al. 2001), and PDZK1 (Chen and Li 2005) are also found at synapses although their postsynaptic localization is unclear. In skeletal muscle, syntrophins *α*1, *β*2 and *γ*2 (Ahn et al. 1996; Alessi et al. 2006) and ERBIN (Huang et al. 2001) are localized to neuromuscular junctions, where NBCn1 is highly concentrated (Damkier et al. 2007). NHERF‐1, NHERF‐2, and PDZK1 are abundantly expressed in breast cancer or cell lines (Ghosh et al. 2000; Stemmer‐Rachamimov et al. 2001), where NBCn1 is responsible for pH regulation (Lauritzen et al. 2010; Boedtkjer et al. 2012). On the other hand, in most epithelial tissues including pancreatic ducts and gastrointestinal tracts, NHERF‐1, NHERF‐2, PDZK1 are localized to the luminal side of the tubule (Lamprecht and Seidler 2006), whereas NBCn1 is localized to the basolateral side (Damkier et al. 2007). Thus, it is unlikely that these three proteins interact with NBCn1 in epithelial cells. Their signal intensities were strong in the array experiments, indicating strong biochemical interactions. However, the signal intensity does not necessarily reflect the biological significance of the interaction. In our screens, harmonin was not detected as an NBCn1 binding partner. The reason for the lack of harmonin/NBCn1 interaction remains unclear. It is possible that the interaction involves more than just a single PDZ domain of harmonin. For example, the interaction may require multiple PDZ domains and/or another region of harmonin, such that assessing only a single PDZ domain alone (as was done in our overlays of the PDZ array) does not result in an interaction of high enough affinity to detect.

The functional importance of NBCn1‐binding proteins is their capability to associate NBCn1 with other proteins (Fig. 2). For example, NBCn1 may cluster with NMDA receptors via PSD‐95 or Chapsin‐111. The NMDA receptors bind to the first and second PDZ domains of PSD‐95 (Kim and Sheng 2004), whereas NBCn1 binds to the third PDZ domain (Fig. 1). Thus, the two proteins do not share overlapping binding domains in PSD‐95. The cluster of NMDA receptors and NBCn1 may be required for the transporter to modulate receptor activity by regulating local pH near the receptors. As described above, NHERF‐I, NHERF‐II, and PDZK1 are abundantly expressed in breast cancer or cell lines, where NBCn1 expression is upregulated by 20–30% compared to that in matched normal breast tissue (Boedtkjer et al. 2012). In addition, ERBIN and syntrophin *α*1 are also abundantly expressed (Liu et al. 2008; Bhat et al. 2011). The NBCn1 interaction with ERBIN is particularly interesting as ERBIN binds to the ErbB2 receptor tyrosine kinase, a key protein playing a role in breast cancer development and progression. Another interesting protein is PDZK1, which has been proposed to serve as a functional regulator for the breast cancer resistance protein in the small intestine (Gorecki et al. 1997). In skeletal muscle, NBCn1 may cluster with signaling proteins at the neuromuscular junction via syntrophins. Syntrophins are the constituents of a large protein complex with dystrophin and dystrophin‐related proteins and also function to localize many signaling proteins such as kinases, ion channels, water channels, nitric oxide synthase to specific intracellular locations (Bhat et al. 2013). Together, the NBCn1 interaction network we constructed is significant in ways that the acid‐extruding protein NBCn1 can cluster with other physiologically important proteins and that such clusters may facilitate their interdependent activities (Kim et al. 1996; Kim and Sheng 2004; Lamprecht and Seidler 2006).

In this study, we examined the interaction between NBCn1 and syntrophin *γ*2 and provided an example of the functional effect of the interaction. Syntrophin *γ*2 binds to NBCn1 via its PDZ domain in both oocyte heterologous expression system and rat brains (Fig. 3). Syntrophin *γ*2 increases NBCn1‐mediated Na/HCO_3_ cotransport (Fig. 4) and ionic conductance (Fig. 5). *I–V* relationships showed an increased slope and a positive shift in zero‐current voltage, reflecting that more NBCn1 proteins are likely expressed in membranes by syntrophin *γ*2. Nonetheless, we note that gamma syntrophins transiently interact with dystrophin in the ER in skeletal muscle and their colocalization is not detected (Alessi et al. 2006). The functional significance of this transient interaction particularly for dystrophin expression in membranes is presently unclear. We think that syntrophin *γ*2 interacts with NBCn1 in the ER and stimulates the transporter. This effect is different from that for PSD‐95 which does not alter NBCn1 cotransport but stimulates its channel‐like activity (Lee et al. 2012). We have proposed a model in which PSD‐95 facilitates dimerization of the two functional NBCn1 subunits and the channel‐like activity is produced by an intermolecular cavity of the dimer (Lee et al. 2012). The model predicts that a regulation of protein trafficking will not separate channel‐like activity from cotransport activity, but instead stimulate both. Thus, the syntrophin *γ*2 effect on NBCn1 is consistent with the model.

In summary, this study shows that NBCn1 interacts with a variety of PDZ proteins, which may allow the transporter to physically and functionally associate with other proteins in a cell‐specific manner. Some of the proteins in the NBCn1 interaction network are known to be involved in diseases, and it will be interesting to investigate the pathophysiology of the interactions in future experiments.

## Acknowledgments

We acknowledge Eunjung Shin for construction of GST/syntrophin *γ*2 fusion proteins, Alana Darcher and Brigid Choi for art work and editorial correction. We also thank Dr. Vincenzo Nigro (Seconda Università degli Studi di Napoli, Italy) for the syntrophin *γ*2 antibody.

## Conflict of Interest

None declared.
